# Parent-driven transmission, off-label SMZ-TMP, and severe pertussis prediction in infants under 2 months: a real-world observational cohort of 527 cases

**DOI:** 10.3389/fped.2026.1815371

**Published:** 2026-09-09

**Authors:** Jieqi Lu, Jikui Deng, Hongmei Wang, Wenhong Zhang, Sen Wang, Lingyun Shao, Shufeng Tian

**Affiliations:** 1 Department of Emergency, Shenzhen Children’s Hospital, Shenzhen, China; 2 Department of Infectious Diseases, Shenzhen Children’s Hospital, Shenzhen, China; 3 Department of Infectious Diseases, Shanghai Key Laboratory of Infectious Diseases and Biosafety Emergency Response, National Medical Centre for Infectious Diseases, Huashan Hospital, Shanghai Medical College, Fudan University, Shanghai, China

**Keywords:** disease transmission, infant, macrolide resistance, pertussis, risk factor analysis, sulfamethoxazole-trimethoprim

## Abstract

**Background:**

Despite high DTaP coverage, pertussis resurgence persists. Infants under 2 months of age face severe outcomes due to vaccine ineligibility and rising macrolide-resistant *B. pertussis* (MRBP), yet treatment options remain limited.

**Objective:**

To analyze epidemiology, clinical profiles, and antimicrobial management of hospitalized pertussis infants under 2 months of age, identifying risk factors for severity and evaluating real-world SMZ-TMP use.

**Methods:**

Retrospective study of PCR/culture-confirmed pertussis infants hospitalized at Shenzhen Children's Hospital (2015–2024). Data included transmission sources, clinical severity, and antimicrobial regimens. Logistic regression analysis was performed to analyze the risk factors for severe pertussis, and a *P*-value < 0.05 was considered statistically significant. The predictive value for severe pertussis was evaluated using the receiver operating characteristic (ROC) curve.

**Results:**

Among 321 infants with a history of exposure, parents constituted the primary infection source (46.1%, 148/321). 25 infants with *Bordetella pertussis* infection (aged 24–59 days) received off-label SMZ-TMP without bilirubin encephalopathy or renal dysfunction. Leukocytosis, and an elevated neutrophil-lymphocyte ratio were predictors of severe disease. Exposure history, apnea, decreased oxygen saturation, tachypnea, tachycardia, elevated white blood cell count, and an increased neutrophil-to-lymphocyte ratio were identified as significant risk factors for severe pertussis.

**Conclusion:**

In infants under 2 months of age with pertussis, parents are the primary source of infection. In infants under 2 months of age with macrolide-resistant pertussis, treatment with SMZ-TMP was effective and no severe adverse reactions were observed.

## Introduction

Pertussis, a highly contagious respiratory infection caused by *Bordetella pertussis*, remains a significant public health challenge despite widespread vaccination. While immunization programs utilizing DTaP vaccines have markedly reduced disease burden, a concerning global resurgence has been observed since the 1990s, even in regions with high coverage such as China (1). This resurgence underscores limitations in current control strategies. Critically, infants under three months of age bear the heaviest burden (2–4): they are too young for vaccination, possess immature immune systems, and are consequently at the highest risk for severe disease, complications (including apnea, respiratory failure, encephalopathy, pulmonary hypertension, leucocytosis), hospitalization, and death. Protecting this vulnerable population is paramount.

The transmission dynamics of pertussis within households are well-recognized, with adults often serving as the primary reservoir due to waning immunity and atypical, easily overlooked symptoms. However, while the general role of adults as sources is established (5), critical knowledge gaps persist regarding the precise identification and characterization of infection sources for infants (≤2 months). Who are the specific household or community contacts most likely to transmit to neonates and young infants? A granular understanding of these transmission pathways is not merely academic; it is essential for designing and implementing targeted interventions, such as optimized “cocooning” strategies (vaccinating close contacts) or enhanced surveillance in specific settings, to effectively shield unprotected infants at their peak vulnerability.

Macrolide antibiotics are the cornerstone of pertussis treatment, aiming to reduce transmission, symptom duration, and mortality through timely administration. Alarmingly, the efficacy of this first-line therapy is increasingly compromised by the global emergence (6) and spread of macrolide-resistant *Bordetella pertussis* (MRBP) since its first report in 1994 (7). Treatment failures associated with MRBP strains complicate management and necessitate alternative regimens. Sulfamethoxazole-trimethoprim (SMZ-TMP) represents the primary alternative for patients over two months old and currently faces less resistance. However, a critical therapeutic dilemma arises for the highest-risk group: infants under two months. SMZ-TMP is strictly contraindicated in this age group due to well-documented risks of renal tubular crystallization and kernicterus (bilirubin encephalopathy), leaving clinicians with no guideline-recommended, effective alternative for MRBP infections in neonates and young infants who are most likely to develop severe or fatal disease.

Paradoxically, in the context of life-threatening MRBP infections in young infants and the lack of approved alternatives, clinicians may resort to administering SMZ-TMP, despite its contraindication in this age group. This real-world clinical practice constitutes a significant deviation from established guidelines, driven by necessity but accompanied by considerable uncertainty regarding the actual benefit-risk profile. Notably, there is a pronounced deficiency of systematic data assessing the real-world effectiveness and, more critically, the incidence and severity of adverse effects associated with this off-label/contraindicated use of SMZ-TMP in infants under two months. Understanding the practical implications of this therapeutic dilemma is crucial to inform urgent clinical decisions, evaluate the true extent of the risk, and potentially guide future guideline revisions or stimulate the development of safer alternatives.

To tackle the dual challenges of protecting and treating vulnerable infants, this study aims to: (1) investigate the specific infection sources of pertussis in infants under two months; (2) analyze clinical characteristics and risk factors for severe pertussis in infants; (3) evaluate current anti-infective therapies, focusing on MRBP infection; (4) assess the clinical outcomes and safety of using SMZ-TMP in infants under two months with MRBP infections, despite its contraindication. These analyses aim to enhance pertussis treatment success, reduce infant mortality, and refine clinical guidelines.

## Methods

### Study design

This was a retrospective cohort study conducted at Shenzhen Children's Hospital from January 1, 2015, to October 31, 2024. The primary objective was to identify risk factors associated with severe pertussis in infants under 2 months of age and evaluating real-world SMZ-TMP use. The secondary objective was to describe the clinical characteristics, complications, and outcomes of hospitalized pertussis patients.

Inclusion criteria: (1) Hospitalized patients aged <2 months; (2) Laboratory-confirmed pertussis by PCR and/or culture between January 2015 and October 2024; (3) Complete medical records available for review.

Exclusion criteria: (1) Incomplete clinical or laboratory data; (2) Patients with confirmed immunodeficiency or congenital heart disease that could confound severity assessment.

Severe pertussis was defined as the presence of any one of the following criteria during hospitalization: (1) Frequent or persistent hypoxemia(SpO₂ < 92% on room air), apnea, or respiratory failure; (2) seizures or encephalopathy suspected to be pertussis-related; (3) echocardiographic evidence of pulmonary hypertension; (4) With one or multiple organ dysfunctions (respiratory, cardiovascular, renal, or neurological); or (5) With an extremely high peripheral blood leukocyte count up to 30 × 10^9^/L which is increasing continuously, accompanied by deterioration of clinical symptoms (8).

### Information collection

Demographic information, underlying medical conditions, clinical signs and symptoms during hospitalization (including paroxysmal spasmodic cough, inspiratory whoop, post-tussive vomiting, cyanosis, tachypnea, wheezing, etc.), complications (such as respiratory failure, renal failure, pulmonary hypertension, pertussis encephalopathy, etc.), treatment details, and prognosis were collected from the electronic medical record information system. Auxiliary examination results were also collected, including complete blood count (white blood cell count, neutrophil count and percentage, lymphocyte count and percentage, neutrophil-to-lymphocyte ratio, etc.), C-reactive protein, blood gas analysis, electrolytes, blood biochemistry, blood culture, chest imaging, cardiac ultrasound, *Bordetella pertussis* culture and PCR results. And within 24 h of admission, sputum specimens (1–2 mL) were collected from the deep respiratory tract of all pediatric patients by trained medical personnel using sterile negative-pressure aspiration for subsequent sputum culture. Additionally, the ResP13 Multipathogen Detection Kit (Registration No. 20183400518), developed by Hybribio, was used for respiratory pathogen detection in nasopharyngeal swab samples. The kit detects 13 common respiratory pathogens simultaneously in a single tube using multiplex PCR and capillary electrophoresis fragment analysis. The 13 respiratory pathogens are influenza A (H1N1 and H3N2), influenza B, respiratory syncytial virus (RSV), adenovirus, parainfluenza, mycoplasma, chlamydia, rhinovirus, metapneumovirus, bocavirus, coronavirus. Only qualified sputum specimens (microscopic criteria: >25 leukocytes per low power field and <10 epithelial cells per low power field) were accepted as representative of the lower respiratory tract. A panel of three experienced pediatricians then evaluated the isolated bacteria using standardized diagnostic protocols (9), integrating all available clinical and laboratory data (e.g., symptoms, imaging, inflammatory markers) to confirm true bacterial co-infection rather than colonization. The minimum inhibitory concentration (MIC) values of *Bordetella pertussis* isolates against erythromycin, and sulfamethoxazole-trimethoprim (SMZ-TMP) were determined using the E-test method.

The general information and clinical data were verified by a pediatric infectious disease specialist with more than 5 years of clinical experience.

### Ethics and informed consent

This study was approved by the Shenzhen children's Hospital Ethics Committee (No. 201907907).

### Statistical analysis

Statistical analysis was performed using SPSS version 27.0 software. Normally distributed continuous variables were expressed as mean ± standard deviation (*x¯* *±* *s*), and intergroup comparisons were conducted using Student's t-test. Non-normally distributed continuous variables were presented as median (interquartile range) [*M (P25, P75)*], with the Mann–Whitney U test employed for group comparisons. Categorical data were expressed as counts, percentages, or constituent ratios (%), with intergroup comparisons performed using: the chi-square test (when *n* ≥ 40 and all expected frequencies T ≥ 5), the continuity correction formula (when *n* ≥ 40 and 1 ≤ T < 5), or Fisher's exact test (when *n* < 40 or T < 1). A two-tailed *P*-value < 0.05 was considered statistically significant.

## Results

### Epidemiological characteristics

**Figure 1:**
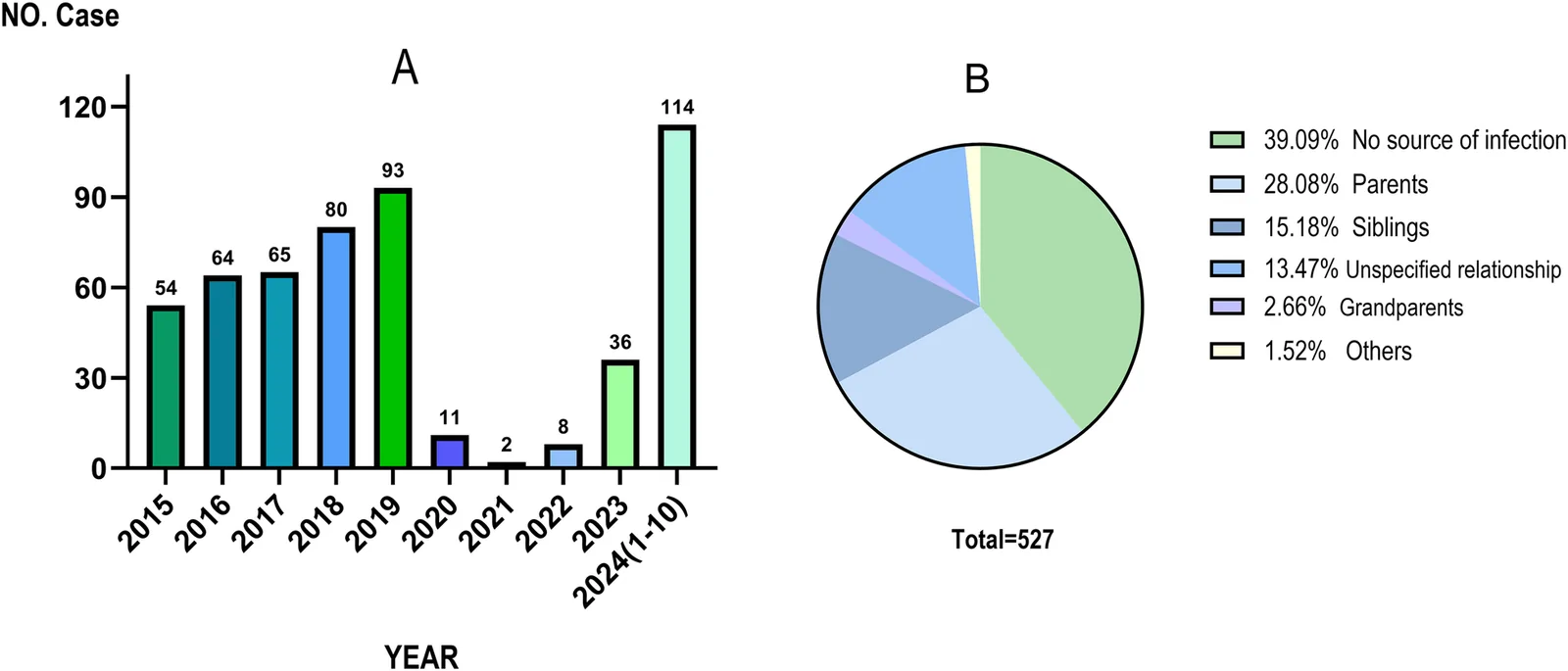
Epidemiological characteristics of pertussis in hospital (*N* = 527) (**(A)** annual case numbers; **(B)** the history of exposure distribution). **(B)** “Unspecified relationship” refers to individuals with a confirmed history of cough exposure but without documented relation to the pediatric patient in medical records. “Others” includes aunts, confinement nannies, domestic helpers, caregivers, neighbors.

This study enrolled 527 hospitalized infants under 2 months of age diagnosed with pertussis at Shenzhen Children's Hospital between January 2015 and October 2024. The cohort comprised 276 males (52.4%) and 251 females (47.6%). From 2015 to 2019, the annual incidence of pertussis demonstrated a progressive increase. A marked decline in case numbers was observed from 2020 to 2022, followed by a rapid resurgence in 2023 (Figure 1A). Among the enrolled patients, 321 cases (60.9%) had definitive exposure history to individuals with cough symptoms. Parental transmission accounted for the majority of exposures (*n* = 148, 46.1% of exposed cases), followed by sibling transmission (*n* = 80, 24.9% of exposed cases) (Figure 1B).

### Clinical manifestations

**Figure 2:**
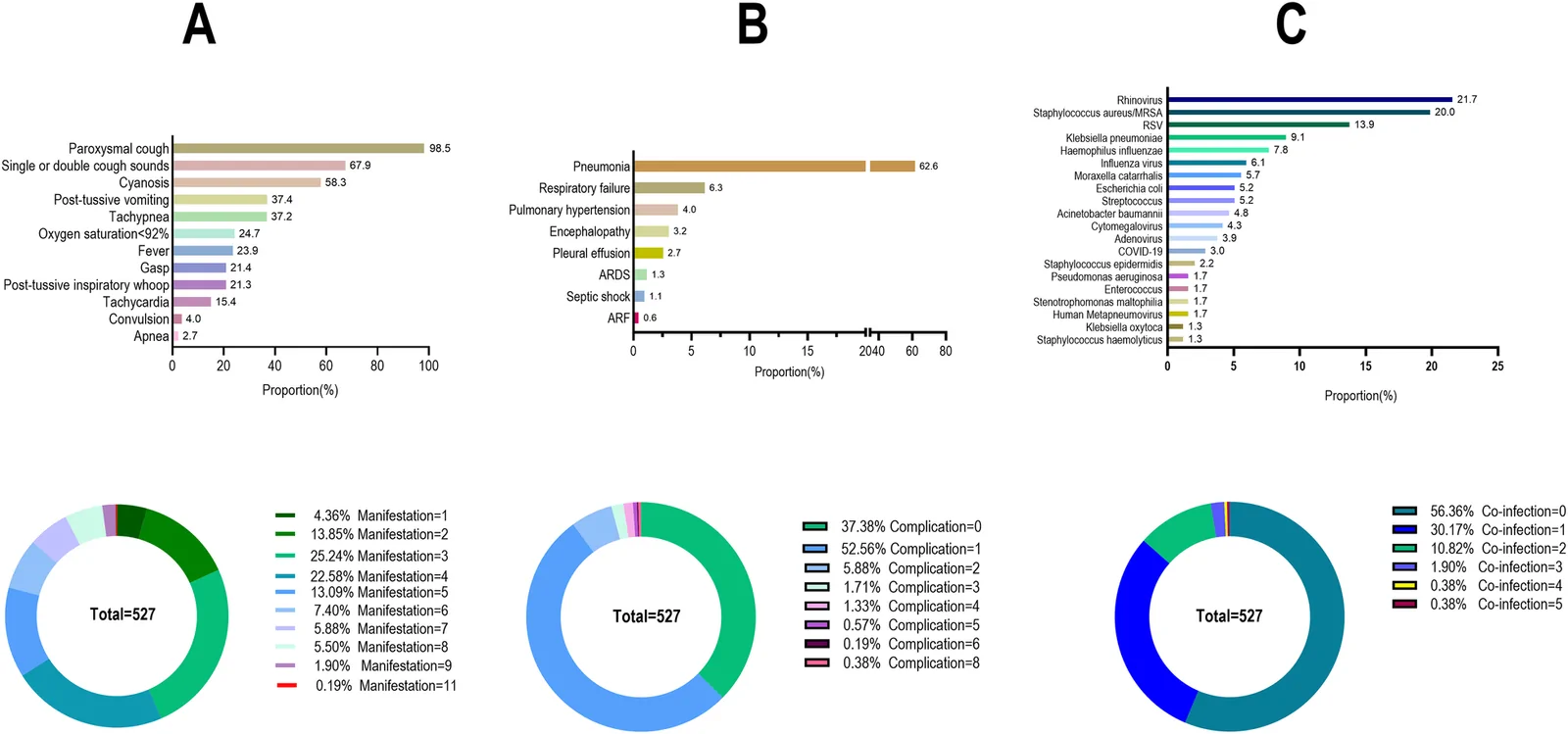
Clinical manifestations **(A)**, complications **(B)**, and co-infections **(C)** (*N* = 527). 0-11 indicates the number of consolidations.

Among the 527 pediatric cases, the three leading clinical manifestations were paroxysmal cough (519 cases, 98.5%), single or double cough sounds (358 cases, 67.9%), cyanosis (307 cases, 58.3%) (Figure 2A).

Among the 527 pertussis-positive cases, a total of 330 pediatric patients developed complications (accounting for 62.6%). The most prevalent complication was pneumonia (*n* = 330, 62.6%), followed by respiratory failure (*n* = 33, 6.3%) (Figure 2B).

A total of 297 cases (56.4%) were infected with *Bordetella pertussis* alone, while 230 cases (43.6%) exhibited co-infection with other pathogens. Among the co-infections, the top three viral pathogens were rhinovirus (50 cases, 21.7%), respiratory syncytial virus (32 cases, 13.9%), and influenza virus (13 cases, 5.7%). The top three bacteria detected were: *Staphylococcus aureus* in 46 cases (20.0%), *Klebsiella pneumoniae* in 21 cases (9.1%), and *Haemophilus influenzae* in 18 cases (7.8%). Furthermore, among the 230 cases in which *Bordetella pertussis* coexisted with other pathogens, infection with two or more additional pathogens was detected in 71 cases, accounting for 30.9% (71/230) (Figure 2C).

### Drug resistance analysis

**Figure 3:**
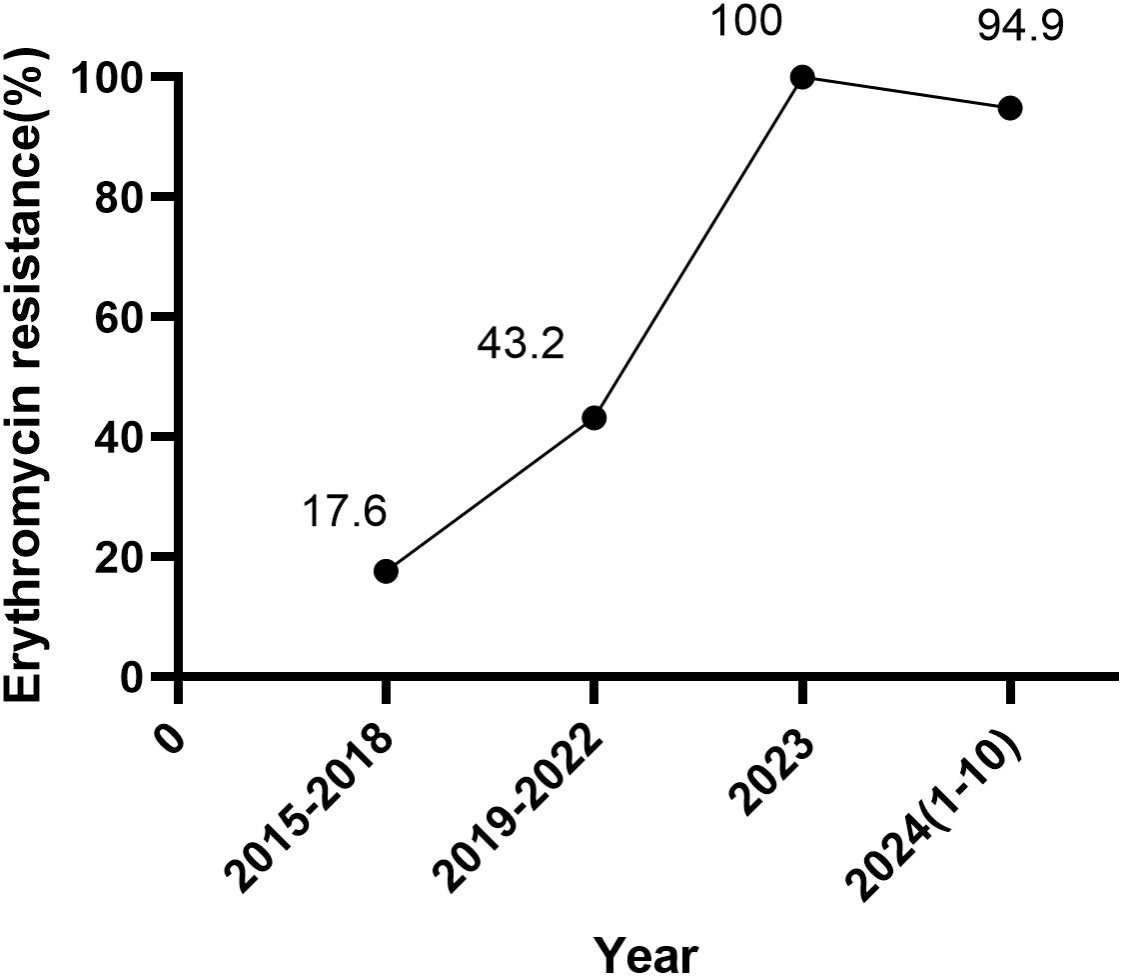
Trends of erythromycin resistance.

From January 2015 to October 2024, antimicrobial susceptibility analysis was performed on 133 *Bordetella pertussis*-positive patients among hospitalized pertussis cases under 2 months of age. Among the *B. pertussis* isolates, 38 were susceptible to erythromycin, while 95 were resistant, resulting in an overall resistance rate of 71.4% (Figure 3).

### Treatment efficacy evaluation

Among 297 patients with *Bordetella pertussis* monoinfection, the antimicrobial regimens were distributed as follows: Macrolide monotherapy: 214 cases (72.1%), Macrolide +β-lactam combination therapy: 55 cases (18.5%), Macrolide + SMZ-TMP: 17 cases (5.7%), β-lactam + macrolide + SMZ-TMP: 7 cases (2.3%), β-lactam + SMZ-TMP dual therapy: 2 cases (0.7%), β-lactam monotherapy: 2 cases (0.7%).

In the anti-infective treatment of 95 cases of MRBP, 92 cases (96.8%) received macrolides as initial therapy, and 34 cases (35.8%) subsequently required switching to sulfamethoxazole-trimethoprim (SMZ-TMP) for anti-infective treatment. Among the 29 cases treated with β-lactam antibiotics (including cefoperazone-sulbactam, piperacillin-tazobactam, ceftriaxone, amoxicillin, and cefaclor) for pertussis, 13 cases (44.8%) were either concurrently administered or switched to SMZ-TMP treatment.

Of 527 infants diagnosed with pertussis, a total of 57 received SMZ-TMP for anti-infective therapy. From 2015 to 2022, there were 7 cases; in 2023, there were 10 cases; and in 2024, there were 40 cases. Among these, 25 cases involved off-label use of SMZ-TMP, for which informed consent was obtained from the guardians. The age of the infants at the time of SMZ-TMP treatment ranged from 24 to 59 days. The dosage administered was sulfamethoxazole 20 mg/kg and trimethoprim 4 mg/kg every 12 h. The treatment duration during hospitalization ranged from 1 to 14 days, with a mean of 7.2 days.

Among the 25 pediatric patients who received off-label SMZ-TMP for anti-infective therapy, gastrointestinal reactions were observed in 2 cases (8%), and elevated transaminases (ALT 230 U/L, AST 181 U/L) occurred in 1 case (4%). The transaminase elevation appeared 14 days after the initiation of SMZ-TMP and improved following hepatoprotective treatment. During hospitalization, no adverse reactions such as aggravated jaundice, bilirubin encephalopathy, oliguria, hematuria, edema, or other signs of renal insufficiency were observed in any patient. All patients improved after treatment and were discharged, with no mortality reported.

### Risk factors for severe pertussis

In the present study, laboratory data obtained during the hospitalization period were utilized. However, the following variables—fever, paroxysmal cough, post-tussive inspiratory whoop, gasp, post-tussive vomiting, apnea, cyanosis, convulsion, oxygen saturation, tachycardia, and tachypnea—were collected at the time of admission.

**Figure 4:**
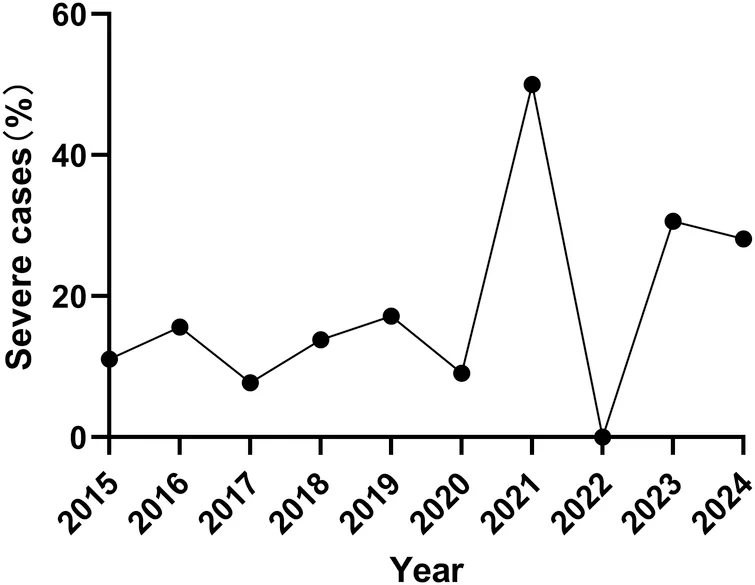
Trends of severe cases.

Based on disease severity, the patients were categorized into a non-severe group (434 cases, 82.4%) and a severe group (93 cases, 17.6%). The incidence of severe pertussis exhibited an upward trend over time (Figure 4). Among the severe cases, the clinical outcomes included survival (87 cases, 93.5%) and death (6 cases, 6.5%). In the deceased cases, the white blood cell (WBC) count consistently exceeded 30 × 10⁹/L. Among these, five cases (83.3%) exhibited a higher neutrophil percentage than lymphocyte percentage. Five cases (83.3%) required endotracheal intubation with mechanical ventilation, four cases (66.7%) underwent exchange transfusion therapy, three (50%) received continuous renal replacement therapy (CRRT), three (50%) were treated with nitric oxide (NO) inhalation, and one case (16.7%) required extracorporeal membrane oxygenation (ECMO) support. More details are provided in Table 1.

**Table 1:** The characteristics of the six fatal cases.

Clinical data	Case 1	Case 2	Case 3	Case 4	Case 5	Case 6
Age (d)	43	38	46	36	36	42
History of DTaP (Y/N)	N	N	N	N	N	N
Year of onset	2017	2019	2023	2024	2024	2024
Symptoms at the time of hospitalization	Convulsion, ARDS, Shock, ARF	Convulsion, Respiratory failure, Shock, ARF	ARDS	ARDS, Shock	Respiratory failure, Shock, ARF	Respiratory failure
Pulmonary hypertension (Y/N)	Y	Y	Y	Y	Y	Y
WBC (×10^9^/L)	101.85	77.59	49.98	44.77	33.02	59.48
Time from onset to hospitalization(d)	4	14	7	8	12	17
Date of onset (YYYY-MM-DD)	2017-02-11	2019-10-24	2023-12-22	2024-01-20	2024-01-31	2024-03-15
Initial treatment (YYYY-MM-DD)	2017-02-17	2019-11-07	2019-12-27	2024-01-28	2024-02-08	2024-03-30
Date of death (YYYY-MM-DD)	2017-03-02	2019-11-14	2024-01-29	2024-02-02	2024-02-15	2024-04-03
MRBP	NA	NA	NA	NA	NA	NA
Date of SMZ-TMP use (YYYY-MM-DD)			2024-01-05 to 2024-01-11			

DTaP, diphtheria, tetanus, acellular pertussis combined vaccine; ARDS, acute respiratory distress syndrome; ARF, acute renal failure; MRBP, macrolide-resistant *Bordetella pertussis.*

Comparison of clinical data between severe and non-severe groups (Table 2):

**Table 2:** Comparison of clinical features, complications between severe cases and non-severe cases.

Clinical data	Non-severe (*N* = 434)	Severe (*N* = 93)	*Z*/*X*^2^	*P*
Male [*n* (%)]	233 (53.7)	43 (46.2)	1.704	0.192
A history of exposure [*n* (%)]	253 (58.3)	68 (73.1)	7.068	0.008
Co-infection [*n* (%)]	175 (40.3)	55 (59.1)	11.026	<0.001
Fever [*n* (%)]	77 (17.7)	49 (52.7)	51.413	<0.001
Paroxysmal cough [*n* (%)]	428 (98.6)	91 (97.8)	0.007	0.934
Post-tussive inspiratory whoop [*n* (%)]	99 (22.8)	13 (14.0)	3.57	0.059
Gasp [*n* (%)]	77 (17.7)	36 (38.7)	19.99	<0.001
Post-tussive vomiting [*n* (%)]	167 (38.5)	30 (32.3)	1.266	0.26
Apnea [*n* (%)]	1 (0.2)	13 (14.0)	50.788	<0.001
Cyanosis [*n* (%)]	228 (52.5)	79 (84.9)	33.085	<0.001
Convulsion [*n* (%)]	4 (0.9)	17 (18.3)	55.861	<0.001
Oxygen saturation [*n* (%)]	47 (10.8)	83 (89.2)	253.442	<0.001
Tachycardia [*n* (%)]	21 (4.8)	60 (64.5)	209.693	<0.001
Tachypnea [*n* (%)]	112 (25.8)	84 (90.3)	136.47	<0.001
Pneumonia [*n* (%)]	246 (56.7)	84 (90.3)	37.028	<0.001
Respiratory failure [*n* (%)]	0 (0)	33 (35.5)	164.287	<0.001
Septic shock [*n* (%)]	0 (0.0)	6 (6.5)		<0.001
Pleural effusion [*n* (%)]	0 (0.0)	14 (15.1)	61.421	<0.001
Pulmonary hypertension [*n* (%)]	0 (0.0)	21 (22.6)	96.251	<0.001
Encephalopathy [*n* (%)]	0 (0.0)	17 (18.3)	76.227	<0.001
ARDS [*n* (%)]	0 (0.0)	7 (7.5)	27.613	<0.001
ARF [*n* (%)]	0 (0.0)	3 (3.2)		0.005
WBC[M (P25,P75), ×10^9^/L]	16.175 (12.4775, 20.9125)	27.09 (17.535, 44.58)	−8.4	<0.001
ANC[M (P25,P75), ×10^9^/L]	3.255 (2.43, 4.5475)	7.59 (4.885, 20.63)	−10.839	<0.001
ALC[M (P25,P75), ×10^9^/L]	11.02 (7.685, 14.93)	14.62 (9.96, 20.72)	−4.432	<0.001
LY%[M (P25,P75), %]	69.6 (62.75, 74.4)	54.4 (38.25, 66.25)	−8.267	<0.001
NLR[M (P25,P75), %]	29.3864 (21.889, 40.4075)	62.7642 (36.6893, 125.0715)	−9.001	<0.001
PLT[M (P25,P75), ×10^9^/L]	516.5 (417.5, 605.5)	503 (369.5, 584.5)	−1.566	0.117
CRP[M (P25,P75), mg/L]	0.499(0.499, 0.5)	1.0(0.499, 10.3)	−6.271	<0.001

WBC, white blood cell count; ANC, absolute neutrophil count; ALC, absolute lymphocyte count; LY%, percentage of lymphocytes; PLT, platelet; CRP, C-reactive protein; NLR, neutrophil to lymphocyte ratio; ARDS, acute respiratory distress syndrome; ARF, acute renal failure.

No statistically significant differences (*P* > 0.05) were observed between the severe and non-severe groups in terms of gender, presence of paroxysmal cough, post-tussive vomiting, Post-tussive inspiratory whoop, or platelet count.

However, the severe group exhibited significantly higher frequencies (*P* < 0.05) of the following factors compared to the non-severe group: A history of exposure, co-infection, fever, gasp, apnea, cyanosis, convulsion, oxygen saturation<92%, tachycardia, and tachypnea. Comorbidities:Pneumonia, respiratory failure, septic shock, pleural effusion, pulmonary hypertension, toxic encephalopathy, acute respiratory distress syndrome (ARDS), and acute renal failure. And elevated white blood cell count, neutrophil count, lymphocyte count, NRL, and C-reactive protein (CRP) levels.

Conversely, the non-severe group showed significantly higher incidences (*P* < 0.05) of lymphocyte percentage compared to the severe group.

**Figure 5:**
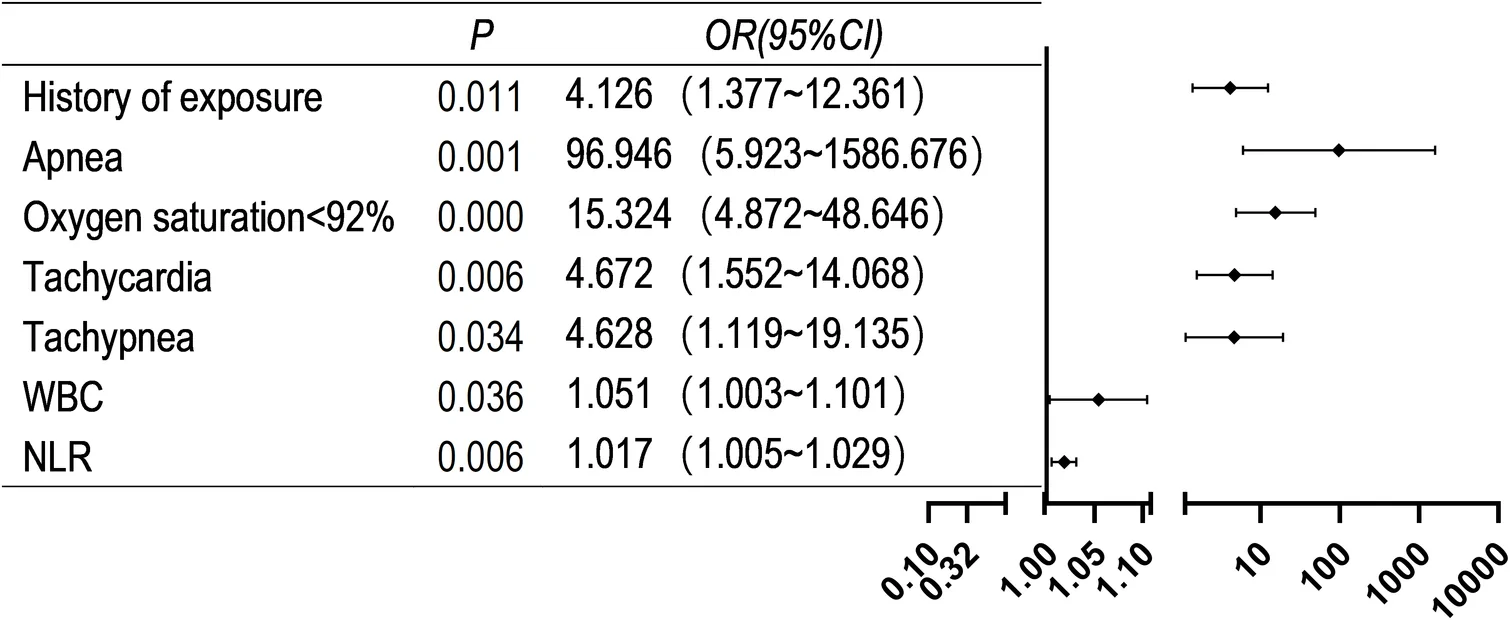
Influencing factors for severe pertussis using multi-variable logistic regression.

Using the progression to severe pertussis as the dependent variable and statistically significant variables from the comparison between the severe and non-severe groups as independent variables, a multivariate logistic regression analysis was performed. The results indicated that significant differences were observed between severe and non-severe pertussis patients in terms of contact history, apnea, decreased oxygen saturation, tachycardia, tachypnea, elevated white blood cell count, and an increased neutrophil-to-lymphocyte ratio (Figure 5).

**Figure 6:**
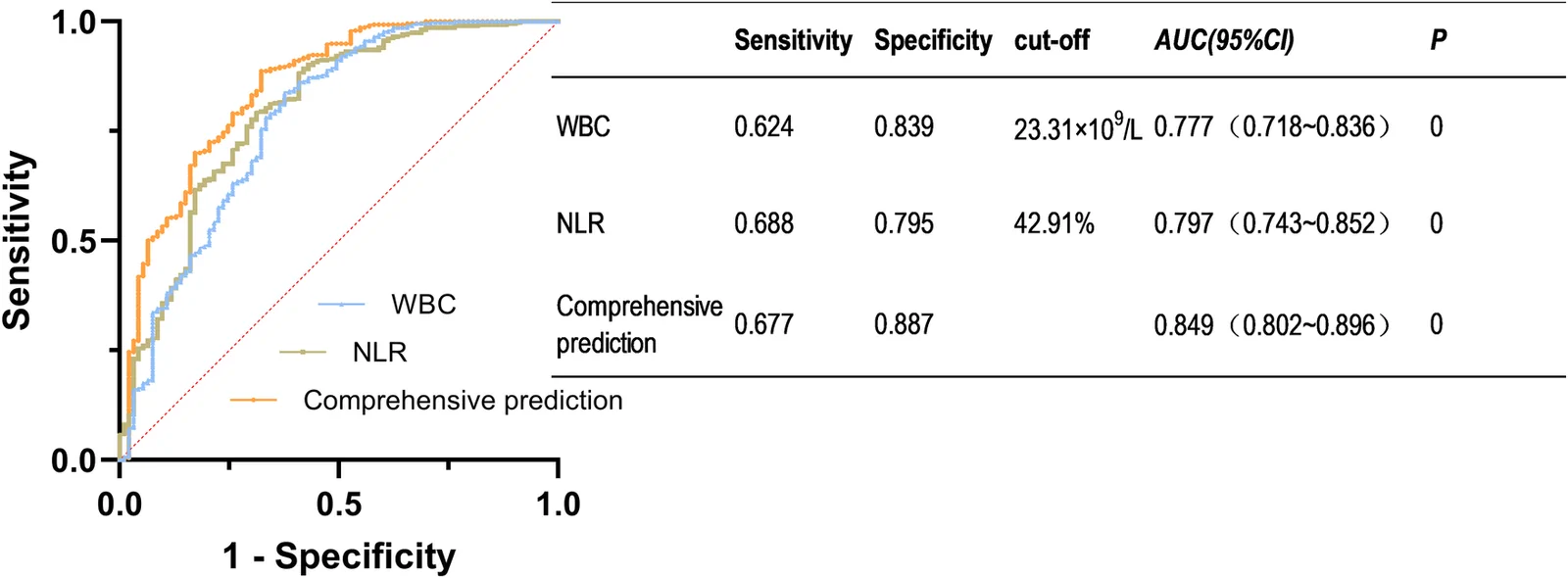
ROC curves of severe pertussis prediction models using multivariable logistic regression. WBC, white blood cell count; NLR, lymphocyte to neutrophil ratio.

When a combination of factors including white blood cell count and neutrophil-to-lymphocyte ratio (NLR) was used to predict severe pertussis, the sensitivity was 0.677, specificity was 0.887, and the area under the curve (AUC) was 0.849 (95% CI: 0.802–0.896) (*P* < 0.05) (Figure 6).

## Discussion

In this study, the reported cases of pertussis exhibited a year-by-year increase from 2015 to 2019, followed by a decline during 2020–2022 and a subsequent resurgence after 2023 (Figure 1A). This trend aligns with domestic and international reports, which may be attributed to the stringent containment measures implemented during the COVID-19 pandemic (1, 10, 11). Previous studies have demonstrated that pertussis is a disease characterized by familial clustering, with adults typically presenting mild or asymptomatic infections that are prone to being overlooked. Infants and young children constitute the primary affected population, whereas adults serve as the major transmission source (5).

In this study, 60.9% of pertussis patients had a clear exposure history, among which parents constituted the largest proportion (Figure 1B). Pertussis is a vaccine-preventable disease; however, neither natural infection nor vaccination confers lifelong protection (12). Under current immunization strategies, the protective antibody levels induced by childhood vaccination decline with age, leading to an increased risk of infection. A Beijing-based study revealed that 70.0% of mothers and 74.7% of neonates had pertussis toxin IgG (PT-IgG) antibody levels below the detection threshold, while those with levels <40 IU/mL accounted for as high as 96.4% and 97.4%, respectively (13). To reduce the risk of infant pertussis, Advisory Committee on Immunization Practices(ACIP) recommends administering the first dose of diphtheria-tetanus-acellular pertussis (DTaP) vaccine as early as 6 weeks of age, and advises pregnant women to receive one dose of tetanus-diphtheriaacellular pertussis (Tdap) vaccine between 27 and 36 weeks' gestation (14).

However, no vaccine protection strategy exists for infants under 2 months of age. Relevant studies indicate that maternal pertussis vaccination during pregnancy provides 31.8% protection for infants aged 2–6 months and up to 80.1% protection for neonates under 2 months of age (15). Research from Switzerland, the UK, the US, and other countries has demonstrated that pertussis vaccination during pregnancy is safe and effective. With the implementation of maternal pertussis vaccination programs in multiple countries, the occurrence rate of severe pertussis in neonates and young infants has significantly declined (16–18). Therefore, to reduce the incidence of pertussis in early infancy, it is essential to enhance awareness of pertussis vaccination among family members. Currently, China lacks an immunization strategy for pertussis vaccination during pregnancy, leaving a gap in preventive measures against pertussis infection in infants under 2 months of age. Further efforts are needed to advance this initiative.

In 2011, macrolide-resistant *Bordetella pertussis* was first identified in asymptomatic school-aged children in China (19). Subsequent studies on *Bordetella pertussis* drug resistance in China have consistently demonstrated high-level resistance to macrolide antibiotics, such as erythromycin, with resistance rates ranging from 36.4% to 97.2% across different regions (20, 21). In the present study, antimicrobial susceptibility testing was performed on 133 clinical isolates of *Bordetella pertussis*, revealing 95 macrolide-resistant *Bordetella pertussis* (MRBP) strains and 38 macrolide-sensitive strains, demonstrating a resistance rate of 71.4%. Moreover, *Bordetella pertussis* was still cultured from the nasopharynx of a substantial number of pediatric patients subjected to erythromycin or azithromycin treatment for over one week (22). Therefore, in the *Guidelines for diagnosis and management and prevention of pertussis of China (2024 edition)*, macrolides are no longer recommended as the first-line anti-infective treatment for patients with pertussis who are macrolide-resistant or for whom empirical macrolide therapy has failed. The guideline indicates that, based on limited data, the efficacy of SMZ-TMP may be slightly superior to that of tetracyclines; the efficacy of macrolides and β-lactams may be comparable. Given the high rate of macrolide resistance in China, for patients with pertussis caused by macrolide-resistant strains, SMZ-TMP should be the preferred treatment for children older than 2 months of age and adults (23).

In our study, following the signing of informed consent for off-label drug use by the guardians of the pediatric patients, SMZ-TMP was administered for anti-infective therapy in pertussis patients under 2 months of age with suspected macrolide resistance. The youngest patient was 24 days old. The results demonstrated clear clinical efficacy, with the most common adverse reaction being gastrointestinal disturbances. No aggravation of jaundice was observed during the treatment course, and no severe adverse reactions were noted. The study by Baskaran Thyagarajan et al. did not find that neonates subjected to oral SMZ-TMP developed kernicterus (24). It is noteworthy that the utilization rate of SMZ-TMP increased significantly in 2023 and 2024. Therefore, under strict adverse reaction monitoring, the feasibility of SMZ-TMP for treating MRBP in this age group warrants further investigation.

In this study, the following factors were identified as significant risk factors for severe pertussis: exposure history, apnea, decreased oxygen saturation, tachypnea, tachycardia, elevated white blood cell count, and an increased neutrophil-to-lymphocyte ratio (Figure 5).

A history of exposure to pertussis is a significant risk factor for severe disease. Identifying this factor enables healthcare providers to detect high-risk patients at an early stage, facilitating the timely implementation of targeted interventions. Such early recognition not only improves patient prognosis, reduces complications, and lowers mortality rates, but also enhances health-care quality and efficiency. Furthermore, it alleviates the economic burden on families and reduces the consumption of public health resources associated with severe pertussis.

An unknown exposure history is often associated with lower exposure intensity and shorter exposure duration, whereas a clear household exposure represents high-dose, prolonged, and repeated pathogen challenge. Previous studies have shown that pertussis patients are more contagious during the catarrhal stage. In household exposure settings, unprotected infants are exposed to high doses of pathogens during the early stage when household members are symptomatic. Consequently, the time required for pertussis toxin to reach its peak level is shortened, and cumulative toxin exposure becomes greater during subsequent continuous contact.

Pertussis toxin increases peripheral white blood cells, primarily lymphocytes. White blood cell aggregation increases blood viscosity. Due to poor deformability, these cells occlude alveolar capillaries, causing microcirculatory disturbance and thrombosis, which may progress to hypoxemia, pulmonary hypertension, hemodynamic failure, and ultimately death (25). A meta-analysis (26) indicated that children with a history of exposure to patients with persistent cough are more susceptible to severe pertussis, potentially attributable to the adult-to-infant transmission mode. Another study demonstrated that when the mother was the sole suspected source of infection, infants experienced earlier onset and more severe symptoms, along with a higher rate of hospitalization. This is hypothesized to result from stronger exposure due to close contact during breastfeeding (27). As demonstrated in our study, the exposed cohort predominantly comprised household members, among whom parents represented the highest proportion. These parents had contact with unvaccinated infants during the early stage of the disease. Infants, in turn, experienced sustained and more intensive household exposure, which was associated with a higher risk of severe disease.

Neonates and infants under 6 months of age are more susceptible to severe symptoms such as apnea following pertussis infection due to their immature immune systems. Frequent apnea episodes can lead to systemic hypoxia, which may result in severe complications, including brain injury, seizures, and even cardiopulmonary arrest, indicating critical illness. Recurrent apnea serves as a prognostic marker for severe pertussis and is significantly associated with mortality (28). In this study, the odds ratio (OR) for apnea was 96.946 (Figure 5), indicating a significantly increased likelihood of severe disease, which is consistent with previous findings.

Among the six fatal cases, pulmonary hypertension was observed in all patients, and the white blood cell count exceeded 30 × 10⁹/L in every case. All cases presented dysfunction of one or more organs, with respiratory system involvement being the most common. The massive aggregation of leukocytes leads to increased blood viscosity, and due to their poor deformability, these cells are prone to obstruct the alveolar capillary bed. This results in localized microcirculatory disturbances and promotes thrombosis, which may further progress to hypoxemia, pulmonary hypertension, and hemodynamic failure, ultimately contributing to death in pediatric patients (25).

The results (Figure 6) demonstrated that a white blood cell (WBC) count exceeding 23.31 × 10⁹/L and a neutrophil-to-lymphocyte ratio (NLR) surpassing 42.91% were associated with a higher probability of developing severe pertussis. Among the six fatal cases, all exhibited WBC counts >30 × 10⁹/L, with five cases (83.3%) showing neutrophil predominance over lymphocytes. Previous studies have identified leukocytosis as an independent risk factor for severe pertussis, with children exhibiting higher neutrophil ratios being more prone to disease progression (1, 29). International researchers comparing fatal versus survived cases of malignant pertussis found that the fatal group had a higher proportion of cases with LC/NC <1 (lymphocyte count/neutrophil count), and WBC elevation >12 × 10⁹/(L·d) was correlated with mortality in malignant pertussis (30). Therefore, close monitoring of WBC counts and NLR is essential during treatment to promptly identify severe disease progression. Timely and aggressive infection management, including exchange transfusion when necessary, may reduce mortality.

Among the six fatal cases, initial treatment was delayed beyond one week after symptom onset in four patients. Previous studies have indicated that the use of clindamycin or azithromycin within seven days of cough onset results in a shorter duration of cough compared to treatment initiated after seven days. Moreover, delayed treatment is associated with an approximately threefold higher risk of respiratory apnea (31). Therefore, early treatment is beneficial for symptom improvement and for reducing the risk of severe disease.

Our study has limitations. First, the retrospective nature of the study and the single-center sampling from a tertiary pediatric hospital may introduce statistical bias. Second, a small subset of patients received preliminary diagnosis and treatment at other medical institutions prior to admission, which may affect the statistical analysis of clinical characteristics. Third, the number of cases involving off-label use of SMZ-TMP is limited, which compromises the representativeness of the findings. Further studies with larger sample sizes are required. Fourth, A limitation of this retrospective study is that laboratory parameters were collected during hospitalization rather than uniformly at admission. Therefore, the identified variables should be interpreted as factors associated with severe pertussis rather than predictors available at the time of presentation. Future prospective studies using standardized admission data are needed to establish early prediction models.

In conclusion, the following factors may increase the risk of severe pertussis progression: exposure history; clinical symptoms/signs (apnea, decreased oxygen saturation, tachypnea, tachycardia); laboratory findings (elevated white blood cell count, increased neutrophil-to-lymphocyte ratio). Leukocytosis, and an elevated neutrophil-lymphocyte ratio were predictors of severe disease. In infants under 2 months of age with macrolide-resistant pertussis, treatment with SMZ-TMP was effective and no severe adverse reactions were observed.

In response to the global resurgence of pertussis, further optimization of immunization strategies in China may enhance protection rates among young infants. Given the increasing resistance of *Bordetella pertussis* to macrolide antibiotics, more effective and safer alternative treatments should be explored.

Enhancing the identification and intervention of risk factors associated with severe pertussis progression in infants under 2 months of age may facilitate early detection, timely treatment, and mitigation of disease progression, thereby reducing severity, shortening disease duration, lowering mortality, and improving prognosis.

## Data Availability

The original contributions presented in the study are included in the article/Supplementary Material, further inquiries can be directed to the corresponding author.
